# Towards a Comprehensive Optical Connectome at Single Synapse Resolution *via* Expansion Microscopy

**DOI:** 10.3389/fnsyn.2021.754814

**Published:** 2022-01-18

**Authors:** Madison A. Sneve, Kiryl D. Piatkevich

**Affiliations:** ^1^Department of Brain and Cognitive Sciences, MIT, Cambridge, MA, United States; ^2^School of Life Sciences, Westlake University, Hangzhou, China; ^3^Westlake Laboratory of Life Sciences and Biomedicine, Westlake University, Hangzhou, China; ^4^Institute of Basic Medical Sciences, Westlake Institute for Advanced Study, Hangzhou, China

**Keywords:** expansion microscopy, optical connectome, super-resolution, electron miscroscopy, synaptic labeling

## Abstract

Mapping and determining the molecular identity of individual synapses is a crucial step towards the comprehensive reconstruction of neuronal circuits. Throughout the history of neuroscience, microscopy has been a key technology for mapping brain circuits. However, subdiffraction size and high density of synapses in brain tissue make this process extremely challenging. Electron microscopy (EM), with its nanoscale resolution, offers one approach to this challenge yet comes with many practical limitations, and to date has only been used in very small samples such as *C. elegans*, tadpole larvae, fruit fly brain, or very small pieces of mammalian brain tissue. Moreover, EM datasets require tedious data tracing. Light microscopy in combination with tissue expansion *via* physical magnification—known as expansion microscopy (ExM)—offers an alternative approach to this problem. ExM enables nanoscale imaging of large biological samples, which in combination with multicolor neuronal and synaptic labeling offers the unprecedented capability to trace and map entire neuronal circuits in fully automated mode. Recent advances in new methods for synaptic staining as well as new types of optical molecular probes with superior stability, specificity, and brightness provide new modalities for studying brain circuits. Here we review advanced methods and molecular probes for fluorescence staining of the synapses in the brain that are compatible with currently available expansion microscopy techniques. In particular, we will describe genetically encoded probes for synaptic labeling in mice, zebrafish, *Drosophila* fruit flies, and *C. elegans*, which enable the visualization of post-synaptic scaffolds and receptors, presynaptic terminals and vesicles, and even a snapshot of the synaptic activity itself. We will address current methods for applying these probes in ExM experiments, as well as appropriate vectors for the delivery of these molecular constructs. In addition, we offer experimental considerations and limitations for using each of these tools as well as our perspective on emerging tools.

## Introduction

### Historical Perspective of Connectomics

Embedded in the concept of the “neuron doctrine” is the principle that neurons communicate through synapses, a striking assumption first made by Ramon y Cajal over a century ago (Ramón y Cajal, 1909). Cajal, in his now-famous illustrations of the silver-stained neurons, was the first person to predict the unique way that neurons are both spatially separated yet connected *via* the synapse (Llinás, 2003; Dhawale and Bhalla, 2008). Cajal was the first to dream of the form of the synapse, but he and his contemporaries were hindered from directly visualizing them by the limitations of the light microscope. The invention of the electron microscope provided researchers with the first toolkit to truly peer at the synapse (Palay, 1956; Wells, 2005). Quickly after its invention, electron microscopy (EM) produced the first high-resolution images of synaptic vesicles, providing key structural evidence for Cajal’s vision of the way in which neurons connect (Robertson, 1953; De Robertis and Bennett, 1955; Palay and Palade, 1955). Sanford Palay, one of the early pioneers of using EM to study the brain, defined the form of the synapse by two common factors: close proximity of the postsynaptic and presynaptic cells divided by a gap of around 200 Å (20 nm), and the presence of mitochondria and vesicles at the presynaptic terminal (Palay, 1956). As microscopy technology advanced, so did the understanding of the structure and form of the synapse.

The modern neuroscientist has the privilege of access to a great deal more knowledge about the structure and function of the synapse than Cajal and his contemporaries would have had. Synapses can be broadly categorized by which of the two distinct mechanisms of synaptic transmission they use—chemical or electrical (Pereda, 2014). Chemical synapses are those which were first visualized through EM and are the more well-studied of the two varieties (Palay, 1956). In chemical synapses, vesicles from the presynaptic neuron release neurotransmitters into the synaptic cleft, which are then recognized by the postsynaptic cell, and thus specific signal defined by a particular neurotransmitter is transmitted. Electrical synapses, on the other hand, transmit information through a fundamentally different means. At an electrical synapse, the communicating cells are physically connected *via* gap junctions, allowing ions, and thus voltage, to be transmitted in most cases bidirectionally between neurons (Pereda, 2014). Electrical synapses were discovered through electrophysiological experiments several years after the first confirmation of the existence of chemical synapses through EM (Watanabe, 1958; Furshpan and Potter, 1959), and their role in the central nervous system (CNS) has only relatively recently been of widespread interest (Gibson et al., 1999; Galarreta and Hestrin, 2001; Hormuzdi et al., 2004). The focus of this review will primarily be on chemical synapses, particularly due to their relative abundance compared to electrical synapses in existing connectomes, although the importance of electrical synapses for brain function should not be underestimated.

Broadly, chemical synapses exist as one of two types—inhibitory or excitatory—based on whether they promote or impede an action potential in the postsynaptic neuron, respectively. In the mammalian CNS, the postsynaptic component of most excitatory synapses and of some inhibitory synapses is located on small protrusions known as dendritic spines (Gray, 1959; Chen et al., 2012; Berry and Nedivi, 2017). Synapses can be further characterized by what neurotransmitter the presynaptic neuron releases, as well as what receptors and scaffold proteins exist in the postsynaptic density (PSD) of spines. For example, the postsynaptic scaffold protein PSD-95, which is expressed only at glutamatergic synapses, is strongly associated with excitatory synapses, and the postsynaptic scaffold protein gephyrin, which interacts with GABA and glycine receptors, is strongly associated with inhibitory synapses (El-Husseini et al., 2000; Prange et al., 2004; Sheng and Kim, 2011). Recent studies have shown that mammalian neurons frequently remodel their spine architecture, assembling and removing excitatory and inhibitory postsynaptic sites in a coordinated manner in response to experience (Chen et al., 2012; Villa et al., 2016). Some individual spines are highly dynamic, appearing and disappearing in a manner of days, while others are more persistent (Berry and Nedivi, 2017).

As the wealth of knowledge surrounding the synapse expands further, there is a need for new technologies that can visualize synapses at high resolution and at high-throughputs. One particularly promising area of study that exemplifies this pressing need is connectomics, the study of wiring of neurons at the resolution of the single synapse. Mapping connectomes in model organisms such as *C. elegans*, *Drosophila melanogaster*, zebrafish, and mice is an immensely difficult and time-consuming endeavor, historically relying on EM. The colossal density of synapses—as many as 1 trillion synapses per cm^3^ of cortex in human brains—combined with the extremely precise resolution needed to visualize single synapses makes the mapping of a connectome a herculean endeavor (Tang et al., 2001; Drachman, 2005). The first whole-organism connectome ever produced was of *C. elegans* hermaphrodite’s 302 neurons and several thousand synapses, which was the result of many years of work from multiple labs and was expanded over time (Albertson et al., 1976; White et al., 1986; Hall and Russell, 1991; Jarrell et al., 2012; Cook S. J. et al., 2019). Recently, the whole-animal synaptic connectome of *Platynereis dumerilii* larva (Verasztó et al., 2020), the partial adult and larvae *Drosophila* connectomes (Ohyama et al., 2015; Scheffer et al., 2020; Hulse et al., 2021), the sea squirt *Ciona intestinalis* connectome (Ryan et al., 2016), a 0.13 mm^3^ volume of the somatosensory cortex of a young adult mouse (Kasthuri et al., 2015), around 1 mm^3^ of mouse visual cortex connectome (MICrONS Consortium et al., 2021), and 1 mm^3^ of the human cerebral cortex (Shapson-Coe et al., 2021) have also been painstakingly reconstructed with EM. Though improvements in EM, such as serial block-face scanning EM, focused ion beam scanning EM, high-throughput serial section scanning EM, and transmission EM, complemented by advanced methods for connectome reconstruction, have facilitated and hastened this process, the imaging of even a partial connectome remains prohibitively demanding of time and resources for most researchers to perform (Xu et al., 2017; Motta et al., 2019; Hubbard et al., 2020; Witvliet et al., 2020). Several ambitious connectomics projects are currently underway, such as the IARPA MICrONS program and the FlyEM project, a multi-lab, multi-year effort which has produced one of the largest and most complete connectomes to date (Dorkenwald et al., 2019; Scheffer et al., 2020; Schneider-Mizell et al., 2020). However, due to the incredible challenge of producing a high-fidelity connectome, only limited volumes of the brain have been mapped so far. This demonstrates the ongoing challenge of imaging synapses at the nanoscale resolution and the need for vast improvements in imaging techniques and technology before connectome reconstruction reaches its full potential.

The speed and resource limitations of traditional methods of connectome reconstruction have significant drawbacks for the usefulness of the connectomes generated. The brain is a highly dynamic structure, and although some synapses and spines are relatively stable, others frequently reassemble, sometimes on the scale of hours (Berry and Nedivi, 2017). A connectome merely represents a snapshot of a brain in a moment in time, and to truly understand the connectivity of an organism, a single connectome will not suffice. Moreover, the connectomes of individual organisms may differ greatly and sometimes unexpectedly (Bergmann et al., 2020; Witvliet et al., 2020). Furthermore, merely knowing the number of synapses that connect two neurons does not provide all of the necessary information for understanding the function of the synapse. The molecular identity of the synapse is incredibly diverse and reveals essential information such as synaptic type and strength, without which a full vision of connectivity cannot be developed, and unfortunately, EM preparation techniques are largely incapable of preserving molecular identity (O’Rourke et al., 2012). On the other hand, optical microscopy is well suited for imaging large samples at high-throughput and compatible with multiplexed imaging required for revealing the molecular identity of synapses. Indeed, high-throughput optical imaging approaches, such as FAST, MOST, and tiling light sheet microscopy, have been already used for whole-brain imaging (Gong et al., 2016; Seiriki et al., 2017; Motta et al., 2019; Winnubst et al., 2019; Chen et al., 2020; Zhong et al., 2021). However, in this case, the resolution is limited by the diffraction of light and thus not sufficient for mapping synaptic connections. Super-resolution microscopy can break the diffraction limit of light but at the cost of greatly reduced throughput and the need for thin sample slicing to maintain point spread function (Sahl et al., 2017; Schermelleh et al., 2019). Expansion Microscopy (ExM), a recently developed tissue processing technique, allows for the imaging of biological specimens at the voxel rates of a diffraction-limited microscope, but with the voxel sizes of a super-resolution microscope (Chen F. et al., 2015; Tillberg et al., 2016). This makes ExM a form of super-resolution microscopy, which relies on swellable polymers to physically expand tissues before imaging (Chen F. et al., 2015). Physical magnification of the specimen occurs at the nanoscale by separating biomolecules, thus enabling subdiffraction limit resolution under a conventional microscope (Tillberg et al., 2016). Here, the authors would like to note that the first method that resulted in brain tissue expansion was reported by Miyawaki and colleagues in 2011 (Hama et al., 2011). However, it was not realized as a way to improve the spatial resolution of imaging until 2015 when Boyden and colleagues introduced the concept of ExM.

ExM has several crucial advantages over EM that make it particularly well suited for visualizing the synapse, particularly for large-scale projects like connectome mapping. For example, the time, labor, equipment, and skill demands of an ExM experiment are substantially less than that of an EM experiment (Wassie et al., 2019). ExM also is compatible with conventional molecular labeling tools and maintains optical microscopy’s ability to image in color, allowing for the use of several fluorescent probes at once or sequentially, thus enabling multiplexing as well as revealing the molecular identity of the synapse *in situ* (Chen F. et al., 2015; Ku et al., 2016; Wassie et al., 2019; Alon et al., 2021; Payne et al., 2021). ExM is compatible with a wide variety of tissue types and has been used to image brain tissue in many of neuroscience’s most widely used model organisms (Freifeld et al., 2017; Gao R. et al., 2019; Yu et al., 2020) and in monkey specimens (Zhao et al., 2017). ExM has already been successfully utilized to image neural connectivity at the resolution of the single synapse. For example, Gao R. et al. (2019) used ExM in tandem with lattice light-sheet microscopy to visualize synaptic proteins and neuronal morphology at nanoscale resolution in the mouse cortex and *Drosophila* brain. Shen et al. (2020) also recently used ExM combined with fluorescent labeling and antibody staining to trace likely synaptic connections in neurons while preserving cell-type specific molecular information. There is tremendous potential for ExM to revolutionize the way synapses are imaged and studied. The technology has produced visually stunning results of brain tissue in a variety of model organisms, and most ExM protocols are substantially more compatible with the high-throughput approach needed to tackle the problems of connectomics and beyond in the future.

We start by reviewing the ExM methods that have been already applied for synaptic mapping and imaging using immunostaining and fluorescent protein-based neuronal labeling and tracing, which facilitates assigning synapses to their parent neurons. We also discuss major challenges and limitations of the currently available ExM methods regarding the comprehensive optical connectome. We then summarize some major molecular strategies for visualizing the synapse at high resolution that can be used in combination with ExM for optical connectome. The first strategy involves fusing synaptic scaffold proteins, such as PSD-95, gephyrin, and synaptophysin, with fluorescent markers. Many tools are variations of this general technique and are widely used both for live imaging and for fixed sample preps, and we feature the most commonly used and the most promising for ExM below. The second strategy involves the use of intrabody-based probes known as FingRs, which bind to synaptic scaffold proteins. Although FingRs are a much newer and less established technology than tagged scaffold proteins, they have several key features that make them more suitable for certain applications. We finalize the review by providing experimental considerations and perspectives on ExM technology.

### State-of-the-Art ExM Methods for the Optical Connectome

All ExM methods are based on physical magnification of biological sample *via* hydrogel embedding followed by mechanical homogenization to disrupt intermolecular complexes (so as to remove mechanical resistance to expanding) and subsequent hydrogel swelling usually in water or low salt buffers. To retain proteins in the expanded state, the sample is treated with a reagent to modify amino acid side chains (usually lysine) with a chemical anchor that participates in radical polymerization to covalently bound proteins into polymer mesh. For a more detailed overview of ExM protocols, we refer readers to recent reviews covering the basic principles of hydrogel-based tissue transformation (Wassie et al., 2019; Choi et al., 2021). As ExM employs optical microscopy for imaging it is heavily reliant on fluorescent probes for targeted biomolecule labeling. Immunohistochemistry (IHC), a fixed tissue staining procedure based on antibody labeling, is a widely used technique within neuroscience (Magaki et al., 2019) and at present, it is the most well-validated approach for synaptic protein visualization using ExM. Since the introduction of the ExM concept in 2014, a large diversity of tissue expansion protocols and methods have been developed for various applications and biological samples. The optimization of ExM was focused on three major aspects: (i) improving fluorescent labeling; (ii) increasing spatial resolution; and (iii) diversifying samples (*i.e.*, whole organs and organisms). We briefly review these aspects in the context of the optical connectome.

**Figure 1:**
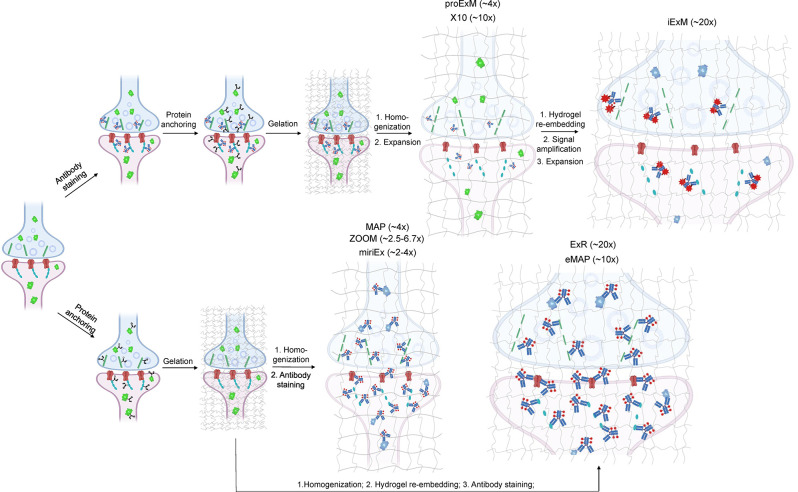
Schematic representations of the ExM workflows with protein retention. Created with Biorender.com.

Depending on the ExM method, antibody staining can be performed either before tissue expansion or after hydrogel embedding and homogenization (Figure 1). In the earlier versions of ExM, fixed tissue is stained with antibodies before it is gelled and expanded, and several particularly useful antibodies for synaptic visualization were demonstrated to be compatible with subsequent tissue expansion (Chen F. et al., 2015; Chozinski et al., 2016). Among the most commonly used antibodies with ExM of brain tissue are Homer and Bassoon (Chen F. et al., 2015; Chozinski et al., 2016; Chang et al., 2017). Staining with antibodies against Homer1 allows for visualization of the post-synaptic components of excitatory synapses in fixed mammalian brain tissue (Gutierrez-Mecinas et al., 2016; Gao R. et al., 2019; Park et al., 2020). Antibodies against Bassoon stain the pre-synaptic components of synapses, and thus are useful to combine with post-synaptic markers like Homer1 (Micheva et al., 2010; Bürgers et al., 2019; Gao R. et al., 2019). Several other important synaptic proteins targeted by immunolabeling with ExM includes glutamate receptor 1, gephyrin, gamma-aminobutyric acid receptor Aα1/Aα2, vesicular glutamate transporter 1, vesicular GABA transporter (vGAT), Rab3A-binding protein (RIM), Shank2, and PSD95 (Chang et al., 2017; Truckenbrodt et al., 2018; Bürgers et al., 2019; Hafner et al., 2019). However, immunostaining of synaptic complexes usually has low efficiency due to the limited accessibility of antibodies to densely packed synapses, where inter-protein distances are smaller than the size of conventional antibodies (Sarkar et al., 2020).

To overcome this limitation, much effort has been focused on the development of ExM methods that allow antibody staining to be performed after sample homogenization (Figure 1). By utilizing mild chemical treatment with the detergent-containing buffer it was possible to preserve antigens for immunostaining in the expanded samples (Ku et al., 2016; Park et al., 2019). In addition, it was demonstrated that tissue expansion provides better access for antibodies to epitopes of the synaptic proteins, which otherwise might be masked in the dense synaptic protein complexes (Sarkar et al., 2020). Molecular de-crowding *via* sample expansion significantly increased the efficiency of immunolabeling, which in turn not only improved visualization of synaptic connections but also expanded the list of commercially available antibodies for synaptic proteins compatible with ExM workflow. For example, the recently developed Expansion Revealing (ExR) method was successfully applied to image 7 synaptic proteins important for neural architecture and transmission including the presynaptic proteins Bassoon, RIM1/2, and the P/Q-type Calcium channel Cav2.1 alpha 1A subunit, and the postsynaptic proteins Homer1, Shank3, SynGAP, and PSD95 (Sarkar et al., 2020). This study also allowed us to gain new insights into the nanoscale alignment of presynaptic calcium channels with postsynaptic machinery in intact brain circuits (Sarkar et al., 2020). Earlier Ku et al. (2016) performed systematic screening of commercially available antibodies for synaptic proteins using ExM modification, called magnified analysis of the proteome (MAP). The screening has further extended the list of synaptic proteins present in different types of synapses (*e.g.*, VGluT1 vs. VGluT2, GABA_B_R1, mGluR5, or different receptors) that can allow detection of synapses coming from different neuron types or brain regions with ExM. The very recent modification of MAP, denoted epitope-preserving MAP or eMAP, was optimized to achieve maximal preservation of antigenicity in mouse and marmoset brain tissue, thus increasing success rates of staining with synaptic antibodies to more than 94% (Park et al., 2021).

Another advantage of post-expansion immunostaining is the ability to carry out multiple rounds of labeling and imaging, providing an unprecedent degree of multiplexed super-resolution synaptic proteomic profiling. When expanded tissue samples are imaged in between rounds of immunostaining and antibody stripping, a variety of antibody signals can be converged into a single composite image, allowing for several synaptic proteins to be stained for and imaged at once. For example, the MAP and eMAP protocols were demonstrated to be suitable for highly multiplexed super-resolution imaging *via* repeated staining and destaining using antibodies for synaptic proteins including Homer1, Bassoon, PSD95, vGluT1, vGluT2, and GABA_B_R1 (Ku et al., 2016). A similar approach was implemented in multi-round immunostaining Expansion Microscopy (miriEX) that involves tissue expansion followed by several iterations of antibody staining and stripping (Shen et al., 2020). The miriEX method was originally applied for iterative immunostaining endogenous synaptic proteins, such as Cannabinoid type 1 receptor, calbindin, and serotonin transporter, in mouse neurons that expressed Brainbow constructs (Shen et al., 2020). While iterative immunolabeling is proven to be a valid approach for multiplexed ExM imaging, it might not be a very practical one due to the need for extremely precise image co-registration between staining steps. Furthermore, this procedure involves buffer exchange and thus it can slightly alter the expansion factor due to osmolarity mismatch. Co-registration of images with varying expansion factors significantly complicates image processing and analysis steps (Alon et al., 2021). Alternatively, multiplexing can be achieved *via* blind unmixing without reference spectral measurements, allowing up to 15 color imaging (Seo et al., 2021). Seo at el. utilized spectral unmixing on expanded brain samples although only combing four labels for NeuN, GFAP, calretinin, NF-H. The multiplexing approach can significantly increase the utility of ExM for molecular profiling of synapses, however, it still needs to be carefully validated for a large variety of synaptic proteins.

**Figure 2:**
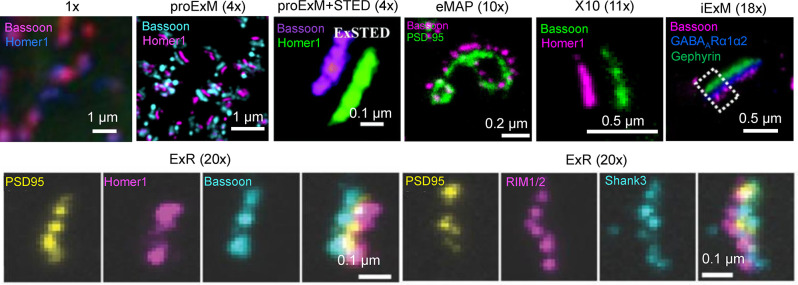
Visualization of synapses in mouse brain tissue using different ExM protocol modifications. The values in parenthesis represent the linear expansion factor for the corresponding image. Adapted from Chen F. et al. (2015), Chang et al. (2017), Li et al. (2018), Truckenbrodt et al. (2018), Gao R. et al. (2019), Sarkar et al. (2020), and Park et al. (2021).

In addition to 3D mapping and molecular profiling of synaptic connections, ExM methods with increased expansion factors can be used to visualize the nanoarchitecture of synapses (Figure 2). The resolution power of ExM is defined by the expansion factor. The majority of the ExM methods enable about four-fold sample expansion in linear dimension, which results in ~70–80 nm of lateral resolution under conventional diffraction-limited microscopes (Tillberg et al., 2016). One strategy to increase the expansion factor is based on the iterative expansion through gel re-embedding, i.e., synthesis of new hydrogel network within an already expanded sample. This strategy was first employed in iterative ExM, or iExM, to achieve ~16–22-fold linear expansion (Chang et al., 2017). The iExM method was carefully validated and characterized by imaging tubulin structures in cultured cells demonstrating isotropic expansion and about 25 nm of effective resolution. The achieved resolution was sufficient to clearly separate post- and pre-synaptic density proteins, such as Homer1, Bassoon, and Gephyrin, from neurotransmitter receptors GluR1 and GABAARα1/α2 in excitatory and inhibitory synapses, respectively (Chang et al., 2017). However, in iExM the proteins are not retained in the expanded state and the staining relies on custom oligonucleotide conjugated antibodies in combination with signal amplification *via* locked nucleic acid probes. Requirements for customized reagents prevented wide adaptation of iExM. Protein retention with the iterative expansion concept was realized in ExR (Sarkar et al., 2020), tetra-gel-based ExM (Gao et al., 2021), and eMAP (Park et al., 2021). For example, ExR, exhibiting effective resolution comparable to that of iExM, is compatible with commercially available antibodies applied to the expanded samples. Visualizing Cav2.1, PSD95, and RIM1/2 in mouse brain tissue revealed how calcium channel distributions participate in transsynaptic nanoarchitecture. Gel re-embedding complicates and extends sample preparation and treatment steps.

Alternatively, the higher expansion factors (>4-fold) can be achieved by modifying hydrogel chemical composition. For example, by utilizing new hydrogel monomers, Truckenbrodt et al. (2018) developed the X10 ExM method characterized by up to ~11-fold expansion in a single expansion step and compatibility with conventional antibodies. The X10 procedure was used for multi-color imaging of the pre-synaptic active zones and the post-synaptic densities *via* Homer1, Bassoon, PSD95, and synaptophysin staining with an estimated effective resolution of about 25 nm. While X10 provides the convenience of single step expansion, the hydrogel it uses has poor mechanical properties in the expanded state and its polymerization requires special conditions. The expansion factor of the common ExM hydrogel can be also enhanced by adjusting the cross-linker molar ratio and monomer concentration (Park et al., 2019; Hu et al., 2020; Damstra et al., 2021). For example, the ZOOM and TREx protocols, characterized by the expansion factor of six-10-fold, were used for synaptic visualization using Homer and Bassoon antibodies (Park et al., 2019; Damstra et al., 2021), although the staining of other synaptic proteins still remains to be tested with these methods. It should be also noted that enhancement of resolution can be achieved by combining sample expansion with traditional super-resolution imaging techniques. For example, ExM was combined with stimulated emission depletion microscopy (STED), stochastic optical reconstruction microscopy (STORM), super-resolution optical fluctuation imaging (SOFI) to gain additional improvement in resolution (Gao et al., 2018; Li et al., 2018; Xu et al., 2019; Zwettler et al., 2020; Shi et al., 2021). However, to date, only ExSTED, a combination of ExM with STED, has been applied for synaptic imaging in mouse brain tissue (Li et al., 2018). The proof-of-principle applications of the ExM protocols with increased expansion factor clearly demonstrated the advantages of enhanced resolving power for synaptic nanoarchitecture imaging. However, further validation and characterization are required for establishing the new protocols for routine use in neuroscience research. In particular, it is important to confirm the distortion-free expansion of synaptic complexes, which are known to be very densely packed with proteins and tight proteins complexes.

The ExM methods reviewed above were mostly optimized for cultured cells or thin brain tissue sections. However, neural processes project to local and distal areas throughout the brain, reaching more than 78 cm in total length in the mouse brain (Winnubst et al., 2019). Therefore, imaging of small brain tissue volumes would have limited utility for complete connectome imaging and reconstructions, as registration of neuronal processes from independently expanded and imaged tissue sections from the same brain may not be feasible. It would be ideal if one could expand and image the intact brain with appropriate synaptic and neuron labeling as well as resolution to form a comprehensive connectome. However, this task meets several technical challenges on multiple fronts—expansion procedure, labeling, and imaging itself, which still remain to be fully addressed in practice. For example, four-fold expanded mouse brain with dimensions of 4 × 3 × 6 cm is impossible to image with nanoscale resolution in intact form due to the limited working distances of available objectives. One way to deal with this is to slice the expanded brain upon imaging, a solution already implemented in fMOST technology (Zhong et al., 2021) and in the MouseLight project (Winnubst et al., 2019).

Reducing the expansion factor of the original ExM protocol can facilitate entire brain imaging. For example, two-fold expanded mouse brain using the CUBIC-X protocol can be imaged under a custom light-sheet microscope, albeit with the cost of greatly reduced spatial resolution (Murakami et al., 2018). On the other hand, expansion of small model organisms such as *C. elegans*, *Drosophila*, and zebrafish, could be more feasible at present, as expanded samples can be imaged in an intact state using conventional imaging setups. ExM methods were already adopted for super-resolution imaging of *Drosophila* brain explant (Mosca et al., 2017; Jiang et al., 2018), enabling the mapping of presynaptic sites in the entire brain with lattice light sheet microscopy (Gao R. et al., 2019; Lillvis et al., 2021). It was also demonstrated that zebrafish brain explants (Freifeld et al., 2017) and even whole zebrafish larvae (up to 6 days post-fertilization; Sim et al., 2021) can be expanded and imaged using conventional imaging setups. Similarly to whole zebrafish expansion (Sim et al., 2021), the ExCel method can expand an entire *C. elegans* specimen using an extensive chemical treatment to ensure isotropic expansion (Yu et al., 2020). However, this treatment reduces the fluorescence of fluorescent proteins and antibodies, requiring a larger amount of antibodies and extended staining time (Yu et al., 2020). To facilitate antibody penetration into expanded samples, stochastic electrotransport (Kim et al., 2015) was shown to speed up antibody diffusion into thick (>5 mm) expanded mouse brain samples (Ku et al., 2016). Immunostaining of magnified samples extends the timeline and increases the cost of sample preparation.

In addition to identifying the synapse itself, an important part of connectomics is tracing synaptic connections to the originating neuron. From this perspective, the expression of fluorescent proteins might be a good alternative to antibodies, as the visualization of genetically encoded fluorescent probes does not require an additional staining step and they can be evenly expressed throughout the plasma membrane and/or cytoplasm. Protein-retention ExM (proExM) was demonstrated to retain native fluorescence of multiple fluorescent proteins in expanded samples, including mouse and monkey brain tissue (Tillberg et al., 2016). Owing to its high brightness and chemical stability, yellow fluorescent protein (YFP) has high performance in ExM and was used for neuronal tracing in mouse tissue (Gao R. et al., 2019). Indeed, cytoplasmic or membranal expression of fluorescent proteins is perhaps one of the most widely used approaches for neuronal tracing using optical microscopy. Fluorescent protein-based technology for neuronal tracing, such as Brainbow, has been already used in combination with ExM (Tillberg et al., 2016; Chang et al., 2017). Brainbow is a transgenic strategy for distinguishing individual cells from their neighbors, due to stochastic fluorescent protein expression that provides individual cells a unique fluorescent signature (Livet et al., 2007; Cai et al., 2013; Weissman and Pan, 2015; Shen et al., 2020). Brainbow allowed for individual neurons to be distinguished from the neighbors in mouse brain slices (Tillberg et al., 2016), while antibodies against gephyrin, Homer1, and Bassoon allowed those synapses to be characterized as excitatory or inhibitory (Shen et al., 2020). Improved versions of Brainbow, such as Tetbow (Sakaguchi et al., 2018), and Bitbow (Li et al., 2021), have been demonstrated to enable highly efficient neuronal morphology reconstruction. Applying proExM to the Bitbow-expressing *Drosophila* brain made it possible to reconstruct all 21 ventral nerve cord serotonergic neurons out of 26 estimated total. However, the fluorescent signal was amplified using immunostaining (Li et al., 2021). In addition, the expression of fluorescent proteins using the rabies virus is a powerful transneuronal tracing technology (Ugolini, 2011; Kim et al., 2016).

### Fluorescent Synaptic Scaffold Proteins

Among the most established strategies we discuss is the fusion of synaptic scaffold proteins with fluorescent markers, which has launched many variations upon the theme of fusing a prominent biologically relevant synaptic protein with a fluorescent protein such as eGFP. The most widely used post-synaptic scaffold proteins involved are PSD-95 (Gray et al., 2006; Cane et al., 2014; Isshiki et al., 2014; Villa et al., 2016) and gephyrin (Craig et al., 1996; Villa et al., 2016), and the most widely used pre-synaptic scaffold protein is synaptophysin (Antonova et al., 2009; Li et al., 2010), and variants of these proteins have been imaged in many of the relevant model organisms used by neuroscientists.

Many of the following tools were developed with *in vivo* imaging in mind, but they are likely also compatible *post-vivo* with ExM in fixed tissue. It is our belief that complementing *in vivo* functional imaging of the synapse with ExM of the fixed tissue presents a powerful opportunity to study the structure and function of the synapse in tandem. Structural and connectomic data are significantly more useful when complemented by functional data: for example, a connectome alone would not reveal the strength of an individual synapse, but a connectome supplemented with functional data would present a much clearer picture of the synapse in question (Turner et al., 2020). ExM is uniquely situated to synergize structural and functional imaging and produce connectomes that are supplemented with custom functional data.

### Probes to Visualize Post-synaptic Connections

We begin by describing tools to visualize the postsynaptic scaffold proteins. These approaches enable investigations of structural dynamics in live brains and provide fluorescent markers of synapses for post-fixed tissue expansion. In many researched vertebrate systems, commonly used proteins for fluorescence tagging are postsynaptic density protein 95 (PSD-95), also known as synapse-associated protein 90 (SAP-90), and gephyrin. These particular proteins are found at the postsynaptic density, a dense protein complex found in both excitatory and inhibitory synapses (Sheng and Kim, 2011; Dosemeci et al., 2016). The postsynaptic density of an excitatory neuron can contain several hundred PSD-95 molecules, and the postsynaptic density of an inhibitory synapse can contain tens or even hundreds of gephyrin molecules (Chen X. et al., 2005; Chen et al., 2011; Choii and Ko, 2015). While PSD-95 and gephyrin are among the most common postsynaptic density proteins studied in vertebrates, the postsynaptic density is full of many other proteins which could alternatively be labeled to provide insight into the structure of synapses (Helm et al., 2021).

**Figure 3:**
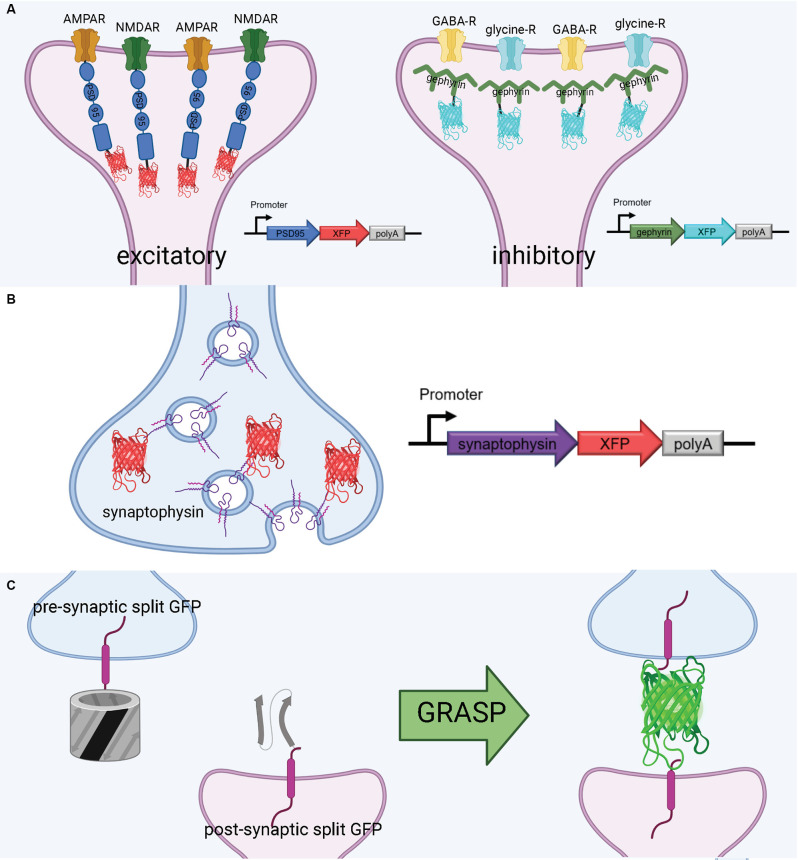
Tools to visualize post-synaptic and pre-synaptic connections. **(A)** Schematic diagram of post-synaptic labeling of excitatory synapses with exogenous PSD-95-fluorescent protein hybrids and inhibitory synapses with exogenous gephyrin-fluorescent protein hybrids. **(B)** Schematic diagram of pre-synaptic labeling off synapses with exogenous synaptophysin-fluorescent protein hybrids. **(C)** Proximate synapse labeling with GFP Recombination Across Synaptic Partners (GRASP). Created with Biorender.com.

PSD-95 plays a variety of roles in the postsynaptic density, most notably binding to key excitatory glutamate receptors and promoting the maturation and strengthening of dendritic spines and excitatory synapses (Chen et al., 2011; Cane et al., 2014; Taft and Turrigiano, 2014; Chen X. et al., 2015). This makes PSD-95 a faithful structural surrogate for excitatory synapses in vertebrate synapses. Genetically tagging PSD-95 with exogenous fluorescent proteins permits *in vivo* tracking of excitatory synapse structural dynamics (Gray et al., 2006; Cane et al., 2014; Isshiki et al., 2014; Villa et al., 2016; Subramanian et al., 2019; Figure 3A). Some of the first experiments to fluorescently label the postsynaptic density in mammalian and zebrafish neurons used a PSD-95-GFP fusion (Craven et al., 1999; Niell et al., 2004). More recently, PSD-95 has been fused to mCherry for imaging of synaptic spines in live mice. For example, using a CPG15/Netrin knock-out mouse and PSD95-mCherry labeling *in vivo*, Subramanian et al. recently found that GPI-anchored CPG15 interacts with AMPA receptors and recruits PSD-95 to transient, unstable spines, leading to stable long-term synapses (Subramanian et al., 2019). This discovery would have been impossible without a structural marker of PSD-95, underscoring the importance of tools to visualize sub-synaptic structures *in vivo*. PSD-95 has been imaged in expanded mouse brain slices using antibodies (Sarkar et al., 2020); applying a similar technique to PSD-95-mCherry may even increase brightness in the expanded slice. Importantly, the coupling of live PSD-95 fusion imaging with later tissue expansion creates new possibilities for interrogating function and connectivity side-by-side. PSD-95 fusions such as PSD-95-mCherry allow for the structural dynamics and function of synapses to be interrogated in live brains and are then compatible with tissue expansion and microscopy to resolve the fine details of synaptic connections in fixed brains.

Gephyrin is a central postsynaptic scaffold found in the vertebrate CNS exclusively at glycinergic and GABAergic synapses (Craig et al., 1996; Tyagarajan and Fritschy, 2014). Gephyrin tagging with fluorescence proteins has been shown to be a reliable method to mark inhibitory synapses *in vitro* (Meier and Grantyn, 2004; Vlachos et al., 2013; Dejanovic et al., 2014) and *in vivo* (Oh et al., 2016; Villa et al., 2016; Figure 3A). The development of a teal-gephyrin as a morphological surrogate for inhibitory synapses revealed that one-third of inhibitory synapses reside on dendritic spines and that inhibitory synapses are clustered and persistently disassemble and reassemble at persistent sites *in vivo* (Villa et al., 2016). It should be noted that the inhibitory synapses on spines appear to be the most dynamic and plastic inhibitory synapses. Three-color imaging using PSD-95, Gephyrin, and a cell-filling fluorophore resolves three spine types: spines without PSD-95, spines with only PSD-95, and spines with both PSD-95 and gephyrin (Villa et al., 2016; Subramanian et al., 2019). Hence, gephyrin is an effective strategy for labeling inhibitory synapses and can be combined with other fluorescent markers.

In smaller brains, such as that of *Drosophila*, anterograde synaptic tracing is another option for mapping neural connections. For example, trans-Tango has been used to trace synaptic connections in neurons in several *Drosophila* brain regions, such as the olfactory and gustatory systems (Talay et al., 2017) and mushroom body (Scaplen et al., 2021). Trans-Tango relies on the Tango assay (Barnea et al., 2008), which is activated when a pan-neuronally expressed presynaptic fusion protein meets a postsynaptic fusion protein (Talay et al., 2017). Trans-Tango exhibits a high signal-to-noise ratio and can be applied to any genetically defined subset of neurons (Talay et al., 2017).

The expression of exogenous synaptic proteins poses the risk of interfering with the normal function of synapses. For example, overexpression of PSD-95 significantly enhances the amplitude of the excitatory postsynaptic current and increases the number of the synapse (El-Husseini et al., 2000; Béïque and Andrade, 2003). To minimize overexpression, the fusion proteins are usually expressed under weak promoters, e.g., the promoter of the polyubiquitin C gene (UBC; Subramanian et al., 2019). An even safer way to label synaptic proteins can be appending the gene of endogenous protein of interest in the genome with the gene of a tag. One approach for labeling synapses without the consequences of relying on exogenous protein expression is Synaptic Tagging with Recombination (STaR; Chen et al., 2014). STaR is a genetic approach which utilizes recombination in Bacterial Artificial Chromosomes to induce the expression of presynaptic and postsynaptic markers. Importantly, these markers can be targeted to specific neurons and are under the control of their endogenous regulatory mechanisms. In *Drosophila*, Chen et al. (2014) were able to concurrently label both presynaptic and postsynaptic markers by taking advantage of different recombination systems, allowing for visualization of synaptic pairs. The authors used this method to successfully visualize synapses in the *Drosophila* visual system, although there is potential for the method to be used in other model organisms and their appropriate recombination systems (such as Cre-Lox in mice; Chen et al., 2014). STaR is compatible with fixed brain preparations and thus holds potential for higher resolution usage in combination with ExM.

Just as STaR allows for synaptic protein tagging in *Drosophila*, there are *in vivo* genome editing strategies based on the CRISPR-Cas9 technology for mice that have been used to tag cellular proteins with epitopes without interfering with endogenous protein expression (Mikuni et al., 2016; Nishiyama et al., 2017). One such high-throughput option for monitoring endogenous synaptic proteins in live mice at single-cell resolution is SLENDR (Mikuni et al., 2016; Nishiyama et al., 2017). This method involves the delivery of the gene-editing machinery of CRISPR-Cas9 to neural progenitors in developing mouse embryos, traditionally through *in utero* electroporation. The CRISPR-Cas9 technology allows the user to label proteins of interest with small epitope tags, such as human influenza hemagglutinin (HA), Myc, and V5, as well as large payloads like monomeric EGFP and spaghetti monster fluorescent proteins (smFP; Viswanathan et al., 2015). Alternatively, Suzuki et al. developed a homology-independent targeted integration (HITI) strategy based on CRISPR-Cas9, which enables robust DNA knock-in in neurons of postnatal mammals *in vivo* (Suzuki et al., 2016). The HITI approach can be implemented using AAV-mediated gene delivery, reaching about 11% of infected neurons in adult mice. More recently, Gao Y. et al. (2019) introduced the homology-independent universal genome engineering (HiUGE) method, characterized by higher efficiency (>20%) and throughput than HITI. The HiUGE method was successfully utilized in neurons in mice to visualize inhibitory postsynaptic density (iPSD) proteome including inhibitory synaptic protein 1 (Insyn1), inhibitory synaptic protein 2 (Insyn2), and Rho GTPase activating protein 32 (Arhgap32; Uezu et al., 2016). Furthermore, by combining HiUGE with retrograde-transported AAV2-retro serotype (Tervo et al., 2016), in the same study Gao et al. performed neural circuit-selective protein manipulations in the well-defined cortico-striatal circuit and the thalamocortical circuit. Alternative CRISPR-Cas9 methods for targeted genomic integration of epitope tags, such as ORANGE (Open Resource for the Application of Neuronal Genome Editing) and TKIT (Targeted Knock-In with Two), were used for targeting PSD95, GluA1, and GluA2 with short epitope tags *in vivo* in mice with an efficiency of 10–16% (Willems et al., 2020; Fang et al., 2021). The ORANGE system was also validated *in vitro* for a variety of synaptic proteins, including voltage-dependent Ca^2+^-channels, Rab3-interacting molecules, Bassoon, Glutamate receptor NMDA 1, etc. Another option for labeling endogenous synaptic proteins is endogenous labeling *via* exon duplication (ENABLED), which has been utilized specifically to label endogenous PSD-95 in mice. This genetic strategy is particularly notable for connectomics because it minimizes protein overexpression and can label a large subset of neurons (Fortin et al., 2014). These techniques and other similar strategies have the potential to be used to label synaptic proteins, image their dynamics in live mice, and then image synaptic connectivity in expanded brain slices.

### Probes to Visualize Pre-synaptic Connections

Much as with postsynaptic proteins, pre-synaptic proteins can be fluorescently labeled to map synaptic connections. A well-established presynaptic protein for labeling synapses, particularly in vertebrates, is synaptophysin, a synaptic vesicle glycoprotein that interacts with the essential synaptic vesicle protein synaptobrevin and is thought to participate in synaptic vesicle release (Wiedenmann and Franke, 1985; Becher et al., 1999). Synaptophysin fused to a fluorescent protein has been used in many circumstances, such as in mice, rat hippocampal neurons, and zebrafish, as a faithful indicator of presynaptic machinery (Meyer and Smith, 2006; Antonova et al., 2009; Li et al., 2010; Figure 3B). Synaptophysin can also be used in tandem with postsynaptic markers, such as PSD-95, to label synaptic connections. Using this approach, Subramanian et al. (2019) could selectively visualize the dynamics of postsynaptic dendritic spines receiving contact from Synaptophysin-labeled presynaptic terminals. In a similar vein, it is also possible to label a postsynaptic marker such as PSD-95 *in vivo*, and later stain the tissue for a presynaptic marker such as synaptophysin to visualize synaptic connections (Broadhead et al., 2016).

A flexible approach to unambiguously map synaptic connectivity is based on GFP Reconstitution Across Synaptic Partners (GRASP; Feinberg et al., 2008; Figure 3C). Here, two nonfluorescent split-GFP fragments (GFP1–10 corresponding to the first 10 β-strands and GFP11 corresponding to the 11th β-strand of the GFP β-barrel) are tethered to pre- and post- synaptic membranes. Fluorescent GFP is reconstituted only when two neurons, each expressing one of the fragments, are tightly opposed through a synaptic cleft. GRASP, though originally developed in *C. elegans*, has utility in many model organisms (Feinberg et al., 2008). GRASP first was used in *Drosophila* in 2009 and has since been widely used and iterated upon (Gordon and Scott, 2009). For example, one of the first enhanced GRASP variants in *Drosophila* was specifically targeted to synapses by fusion of the presynaptic GFP fragment with Neurexin, a presynaptic cell adhesion protein (Fan et al., 2013). A later variant combines synaptobrevin and GRASP. The syb:GRASP fly chimera allows for *in vivo* activity-dependent circuit mapping, in contrast with the activity-independent neurexin variant (Macpherson et al., 2015). The same team behind the syb:GRASP fly also developed yellow and cyan GRASP variants for *Drosophila*, offering the advantages of multicolor labeling. A further enhancement of GRASP in *Drosophila* is t-GRASP, an activity-independent label which boasts greater signal specificity to the synapse (Shearin et al., 2018).

A mammalian version of GRASP (mGRASP) has had similar success in mapping synaptic connections in mouse brains (Kim et al., 2012; Song et al., 2018). Several variants and enhancements exist, such as CRASP, a cyan fluorescent protein-based variant (Li et al., 2016). Choi et al. (2018) have developed an enhanced GRASP construct, known as eGRASP, which has greater signal intensity than its predecessor. Furthermore, eGRASP expresses either a yellow or cyan fluorescent protein in presynaptic neurons, which allows for visualization of two distinct presynaptic populations which converge on the same postsynaptic neuron.

Neurexin, the presynaptic protein which was targeted in some GRASP variants (Fan et al., 2013), is another synaptic marker of interest, particularly in invertebrate systems (Kim and Emmons, 2017). For example, in *C. elegans*, neurexin located in the presynapse was shown to have an essential role in the development of the morphology of postsynaptic GABAergic neurons (Philbrook et al., 2018). In particular, neurexin is critical for the development of the spine-like protrusions that appear in *C. elegans* neurons (Philbrook et al., 2018). Presynaptic neurexin, in conjunction with the postsynaptic marker neuroligin, can be labeled to define synapses in a directional manner, as was demonstrated in live worms in the iBLINC system (Desbois et al., 2015). iBLINC fuses a recombinant biotin ligase with presynaptic neurexin and a small acceptor peptide with postsynaptic neuroligin. In an iBLINC synapse, biotin is transferred from the presynaptic biotin ligase to the postsynaptic acceptor peptide, and when streptavidin fused with fluorescent protein enters the space surrounding the synapse, it fluorescently labels the postsynaptic biotin, and thus the directional connection between the synapses can be observed (Desbois et al., 2015). Although this system was designed with *in vivo* use in mind, it represents a potential strategy to map synaptic connections in expanded animals, as protein retention expansion microscopy is compatible with streptavidin detection (Tillberg et al., 2016).

Another presynaptic marker used specifically in *C. elegans* is SYD-2, which has been shown to localize exclusively at the presynaptic active zone of worm neurons (Yeh et al., 2005). A fusion protein of SYD-2 and GFP allowed presynaptic puncta to be labeled in live *C. elegans* (Yeh et al., 2005). Furthermore, synaptic protein-protein interactions can now be probed in worms with Turbo ID, an enzyme-based proximity labeling strategy (Branon et al., 2018; Artan et al., 2021). TurboID was used to identify protein-protein interactions that a presynaptic protein, ELKS-1, was involved in Artan et al. (2021), representing a blueprint for a potential strategy for synaptic proximity labeling in *C. elegans*.

An alternative method of visualizing synaptic connections involves genetically-encoded fluorescence-based synapse labeling reagents (Kuljis et al., 2019). Kuljis et al. (2019) recently developed a system which utilizes neuroligin-1, a postsynaptic tag, to target fluorogen-activating protein (FAP) to postsynaptic sites (FAPpost). FAPpost emits a far-red signal upon binding of a small molecule fluorogen, which is applied to brain slices to induce fluorescence. The authors were able to identify synapses belonging to identifiable cell types in the mouse somatosensory cortex. Notably, unlike other constructs for visualizing synapses, FAPpost did not alter synaptic density or neuron firing properties. However, the sparse labeling shown with this technique may not be ideal for assembling complete connectomes.

### FingRs

**Figure 4:**
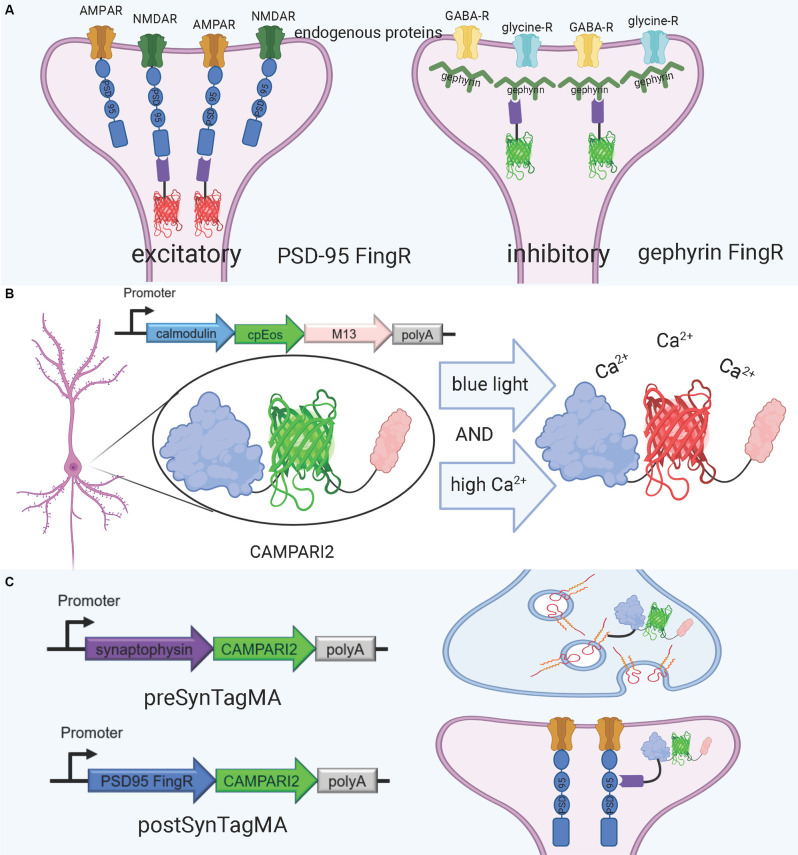
FingRs and related tools. **(A)** Schematic diagram of FingRs for post-synaptic labeling of excitatory and inhibitory synapses, using endogenous PSD-95 and gephyrin, respectively. **(B)** Schematic diagram of CAMPARI2, a genetically encoded calcium indicator, which changes fluorescent wavelength in response to combined blue light and high calcium ion levels. CAMPARI2 forms the fluorescent component of SynTagMA. **(C)** Schematic diagram of pre-SynTagMA, which marks synaptophysin, and post-SynTagMA, which marks endogenous PSD-95 with the aid of a PSD-95-specific FingR. Created with Biorender.com.

An alternative approach for visualizing synaptic proteins is the use of Fibronectin intrabodies generated with mRNA display (FingRs). These are intrabodies fused to fluorescent proteins which bind to the target endogenous protein and allow for precise visualization of the target’s localization and density (Gross et al., 2013; Figure 4A). FingRs are a newer and less established technology than hybrid fluorescent proteins, and they hold great potential for imaging the synapse despite their recency and lack of widespread use. Unlike many other tools, there has been evidence to show that FingRs do not significantly affect endogenous protein expression, the number and strength of synapses, and synaptic transmission in brain slice (Gross et al., 2013; Bensussen et al., 2020). FingRs are expressed in behaving animals and then quantified using post-mortem histology, and must be expressed in living animals for weeks before they are visualized (Gross et al., 2013; Bensussen et al., 2020). This approach can easily be synthesized with existing ExM protocols. However, unlike standard fluorescent protein techniques, FingRs are not compatible with live imaging.

FingRs for PSD-95 and gephyrin were the first-ever developed (Gross et al., 2013), and have since proven useful for visualizing changes in synaptic strength in model organisms. Son et al. (2016) used FingRs in live zebrafish and found that FingR expression did not hinder protein expression at the synapse, number of synapses, and synapse function. Additionally, they used FingRs for PSD-95 and gephyrin to show that chronic hypoxia decreases the number of dopaminergic synapses (Son et al., 2016). Cook S. G. et al. (2019) used FingRs to simultaneously image PSD-95, gephyrin, and CaMKII in the hippocampal neural culture and found that amyloid beta (Aβ) interferes with CaMKII activation in stimulus-induced LTP, but not LTD. Xue Han’s group has recently developed further FingR variants, including a red variant which can be used in conjunction with green variants (Bensussen et al., 2020).

A promising tool for understanding the relationship between structure and function in neural circuits is the recently-developed SynTagMA, a genetically encoded sensor for synaptic activity. SynTagMA is a fusion of a modified version of CAMPARI2, an established indicator of active neurons, with FingRs for either PSD-95 or synaptophysin (Moeyaert et al., 2018; Perez-Alvarez et al., 2020; Figures 4B,C). The PSD-95 SynTagMA construct targets post-synaptic terminals, and the synaptophysin SynTagMA construct targets pre-synaptic terminals (Perez-Alvarez et al., 2020). SynTagMA can detect synaptic activity by irreversibly changing from green fluorescence to red in the presence of calcium upon photoactivation by 395–405 nm illumination, generating a snapshot of synaptic activity at a user-defined time (Perez-Alvarez et al., 2020). SynTagMA can simultaneously tag thousands of active synapses. PSD-95-fused SynTagMA has successfully been used in awake head-fixed mice to visualize active synapses, and future experiments using this synaptically localized, photoconvertible calcium sensor will enable further study on the synaptic basis of complex brain function in health and disease. SynTagMA, if used in conjunction with other tools that map connectivity more directly, could be a crucial tool for understanding functional connectivity.

### Experimental Considerations

**Figure 5:**
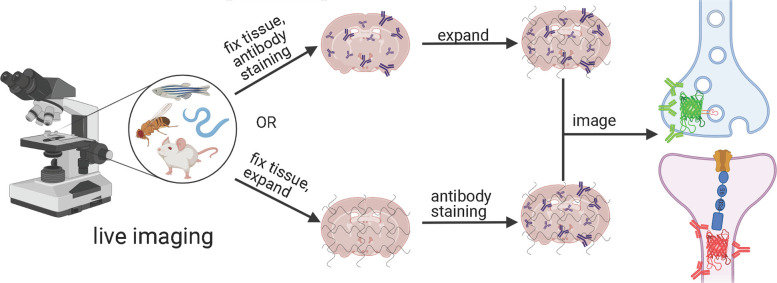
Generalized workflow for combining tools to exogenously label post-synaptic and pre-synaptic neurons in a variety of model organisms with expansion microscopy. Created with Biorender.com.

The tools described in this article are all hypothetically compatible with standard ExM protocols and represent a mere subset of the possible ways to image the synapse at nanoscale resolution. Any imaging or staining done pre-expansion, including live imaging, will proceed normally (Figure 5). Afterward, the tissue intended for expansion can be chemically treated according to your ExM protocol of choice (Karagiannis and Boyden, 2018; Wassie et al., 2019). Though some troubleshooting may be involved to receive optimal results, combining previously established synaptic markers with ExM represents exciting possibilities for synaptic mapping in a variety of model organisms. There are, however, a few caveats to be noted about ExM. For example, while the isotropy of the tissue is generally very well preserved in various tissue types, it is important for new ExM users to validate that their tissues expanded in an isotropic manner (Wassie et al., 2019). However, if the protocol is executed correctly, there is no significant rearrangement of the synapse shown when expanded: the relative position of synaptic proteins stays the same after expansion (Zhao et al., 2017; Sarkar et al., 2020). Although the ExM process can degrade some proteins, fortunately, fluorescent proteins are relatively resistant to degradation during the protocol and maintain their brightness (Wassie et al., 2019). Brightness can be improved by processing the tissue with antibodies prior to expansion and choosing ExM protocols that are best equipped to preserve protein integrity suited for individual project needs (Parra-Damas and Saura, 2020). Another important consideration, specifically when the nanoarchitecture of synapses is studied, is the possible distortion upon expansion that may happen at the nanoscale, which is to be validated for every newly developed ExM method or protocol. The gold standard in the field for synapse identification is EM, but ExM views of synapses cannot be correlated with the ultrastructure as seen in EM, as ExM techniques are not compatible with the staining and sample processing done for EM.

The choice of gene delivery vector is an important consideration for mapping synaptic connections. One important limitation of many of the tools above, especially several of the sensors designed for use in mammalian brains such as mGRASP and SynTagMA, is that they are typically delivered to live mouse brains *via* AAV injection. Although AAVs are the favored gene delivery vector for many experiments, this strategy does not guarantee that every single neuron in the target area is labeled (Chan et al., 2017; Wang et al., 2018). For example, when tracing synaptic connectivity in a small volume of the mouse brain, AAVs delivering Brainbow transgenes were not able to label every neuron for connectivity tracing, and the percent of neurons labeled varied widely based on cell type (Shen et al., 2020). For most applications, it is usually not necessary to label every single neuron, but when assembling complete connectomes, it is essential that as many neurons as possible be labeled. Thus, gene delivery strategies must be applied carefully and cautiously to avoid leaving out unlabelled neurons, particularly in mammalian systems. Alternate gene delivery methods for mice, such as the generation of transgenic mouse lines or herpes simplex virus vectors, which have genomic integration rates much closer to 100%, should be considered for connectome-specific applications.

### Conclusion/Perspective

There is a great need for the generation of connectomes. The first-ever complete connectome of an organism, the *C. elegans* connectome, has been indispensable for studying the worm brain. For example, the worm connectome has been combined with ablation experiments to generate circuit maps and has provided the basis for a number of computational models. Taking inspiration from connectomes has also led to biologically-informed innovations in machine learning (Hasani et al., 2020). However, much of the potential of the connectome has remained locked away, particularly because a single connectome cannot possibly represent the full range of connectivities in even a simple nervous system. Connectomes will be most useful when there are multiple, even hundreds, for a single species, let alone for different sexes, developmental stages, and mutants. Furthermore, many of the most commonly used model organisms, particularly zebrafish, mice, and rats, lack anything resembling a complete connectome, and current endeavors to generate these connectomes, though heroic, are incredibly expensive and time-consuming.

ExM has the potential to represent a paradigm shift in connectomics so that any lab with standard microscopy equipment can contribute to the endeavor to map synaptic connections. Rapid advances in synaptic imaging tools and in ExM protocols have paved the way for a powerful synergy: now all that is left is to turn the hypothetical into reality.

## Author Contributions

KP: conceptualization, supervision, and funding acquisition. MS: writing—original draft preparation. KP and MS: writing—review and editing, visualization. All authors contributed to the article and approved the submitted version.

## Conflict of Interest

The authors declare that the research was conducted in the absence of any commercial or financial relationships that could be construed as a potential conflict of interest.

## Publisher’s Note

All claims expressed in this article are solely those of the authors and do not necessarily represent those of their affiliated organizations, or those of the publisher, the editors and the reviewers. Any product that may be evaluated in this article, or claim that may be made by its manufacturer, is not guaranteed or endorsed by the publisher.
